# Defining and Measuring Sexual Consent within the Context of University Students’ Unwanted and Nonconsensual Sexual Experiences: A Systematic Literature Review

**DOI:** 10.1177/15248380221147558

**Published:** 2023-01-14

**Authors:** Ngozi Anyadike-Danes, Megan Reynolds, Cherie Armour, Susan Lagdon

**Affiliations:** 1 Ulster University, UK; 2 Queen’s University Belfast, UK

**Keywords:** sexual consent, university students, sexual victimization, measurement, definition, systematic review

## Abstract

Lack of sexual consent forms the foundation of unwanted (and nonconsensual) sexual experiences (USEs), yet research suggests it is not well understood amongst university students. While the prevalence of USEs has been well documented within the university context, less is known about how sexual consent is defined or measured. This review aims to identify a consistent sexual consent definition and how current research examining USEs defines and measures sexual consent amongst university students. A systematic review of nine electronic databases (2000–2022) was conducted, and the results were assessed against inclusion criteria (e.g., studies had to focus exclusively on university students). Thirty-three articles were identified and reviewed against the study aims. Sexual consent was more often implicitly defined across measures and articles. Four themes were identified (incapacitation, use of force, use of threats, and lack of wantedness) across the implicit definitions but varied by study with some implicitly defining sexual consent within the context of a relationship. Only three studies explicitly defined sexual consent, referring to it as a willingness to engage in sexual behavior. Measures assessed sexual consent communication or, attitudes and behaviors that might predict sexual aggression. Two studies examined students’ individual sexual consent conceptualizations. Sexual consent appears to be contextual so future research should examine the variability of sexual consent in student samples. Students may indeed rely on implicit sexual consent definitions (rather than explicit) but more research is needed. Lastly, researchers should take care to be clear on their sexual consent definitions, both in text and within measures.

Sexual consent typically denotes some form of agreement to engage in sexual activity; each person can agree (e.g., not incapacitated; Breiding et al., 2016) and there has been no threat, force, or coercion to encourage agreement. Defined in this manner, stripped of any sociocultural and/or legal context, sexual consent does not appear to be so complex. However, sexual consent is nuanced and, thus, must be discussed contextually (Beres, 2007; Levand, 2019). Research suggests that sexual victimization among university students is highly prevalent (Camp et al., 2018; Muehlenhard et al., 2017; Smith, 2010)—approximately 20 to 25% of students have indicated experiencing an unwanted sexual experience (USE) while attending a U.S. university (Kilpatrick et al., 2007; Krebs et al., 2007). The current review will examine sexual consent within the context of university students’ unwanted (and nonconsensual) sexual experiences^1^ as this plays a key part in understanding sexual victimization.

Typically, legal definitions of sexual consent comprise three components: expressed agreement, capacity, and freedom to consent (Kruttschnitt et al., 2014). However, variations exist, particularly in relation to implied consent (which relates to expressed agreement as it is consent inferred from [in]action or certain circumstances), the legal age of sexual consent (which relates to capacity), and coercive behavior (which relates to freedom to consent; Kruttschnitt et al., 2014). To that end, it is useful to define sexual consent within a legal context because the geographical origin of the research is likely to have a set legal framework, and sexual consent knowledge may have been partially sourced from this framework. Nevertheless, research suggests that there is significant variation in the extent to which university students use the law to conceptualize sexual consent (or non-consent^2^) as such it is important that sexual consent be clearly defined (Beres, 2014; Hirsch et al., 2019; Marg, 2020).

Two relatively comprehensive reviews of sexual consent were published recently to consolidate what is currently understood about sexual consent. Muehlenhard et al. (2016) focused on the communication and interpretation of sexual consent between university students, particularly in relation to campus sexual violence. This review contextualizes the issue of USEs, particularly in the U.S. political context, and then briefly outlines the prevalence of USEs on campus and the specific features of university life that may make consent negotiation more complicated (e.g., party culture, alcohol). Their review is extensive and detailed so, briefly, they outline three primary sexual consent conceptualizations: an internal state of willingness (non-observable), an explicit agreement (a direct verbal or written statement), or as behavior interpreted as willingness by someone else (implied or inferred). Ultimately, they conclude that sexual consent can be conceptualized in a multitude of ways and that specific individual differences (e.g., gendered expectations) may impact conceptualization and this, in addition to the college environment, may have a trickle-down effect to communication and interpretation.

Further research by Beres (2014) explored definitions of sexual consent across two datasets. The participants defined sexual consent in three ways: as the minimum requirement for appropriate sex, a discrete event, and as an unrelated feature to the relationship. Beres (2014) stressed that participants’ understanding of sexual consent was distinct from their understanding of communicating willingness to have sex and highlighted the need for explicit language in sexual violence prevention education and research. The second definition has been previously identified in research (e.g., Beres, 2007; Humphreys, 2000, 2004) and has been included within measurements of consent but the last definition is troubling because it suggests that once the relationship has been established, there is no further need to discuss sexual consent. Considering sexual consent as a discrete event rather than a continuous exchange is associated with attitudes and beliefs relating to sexual consent, much of which has been explored within research addressing rape myths related to marital rape (Ferro et al., 2008). Higher rape myth acceptance (RMA) may be related to certain gendered, heteronormative beliefs that men are entitled to sexual intercourse and women must submit to this. Hence, it seems likely that RMA plays a role in an individual’s comprehension and/or conceptualization of sexual consent.

Fenner’s (2017) review highlighted how researchers differ in their definitions of consent and that this definition is often implicitly stated (if at all). Ultimately, Fenner (2017) concluded that sexual consent is defined according to several categories (communication, wantedness or desire, and sexual violence) depending on the focus of the research. More recently (see Marcantonio & Jozkowski, 2021 for review), research has suggested that consent may also be conceptualized as “freely given” (voluntary) or as something that is negated by substance use. Though both these reviews were comprehensive regarding their chosen topic, it is still not apparent how individuals or, more specifically, students, define consent in their own minds and how that definition impacts their actions.

It seems within the research context, sexual consent definitions typically can be divided into two types: implicit and explicit definitions (Fenner, 2017). Explicit sexual consent definitions are stated clearly by the researchers in the body of the text and, if measures are used, the definition will be stated here too. For example, Hickman and Muehlenhard (1999) define sexual consent as “the freely given verbal or nonverbal communication of a feeling of willingness to engage in sexual activity” (p. 259). In contrast, implicit definitions are inferred within the body of the text and may be implied in the context of the measures used. For example, use of language such as “nonconsensual” and “without your consent” in place of a definition. As such, the use of implicit sexual consent definitions encourage both readers (and participants) to use their own definitions of sexual consent, but these personal definitions are not measured. Given that sexual consent is often measured using different and/or nonstandardized measures (Beres, 2014), a lack of clarity regarding researchers’ working sexual consent definitions only further compounds the ability to compare and/or generalize findings (Beres, 2007; Fenner, 2017). Pugh and Becker (2018) took this further in the concluding remarks of their review—academics must come to a consensus on how sexual consent is defined to enable us to best understand how to tackle campus sexual assault.

## Sexual Consent Measures

While some researchers have examined what sexual consent is, others have focused on how to measure it. The Internal Consent Scale and the External Consent Scale (Jozkowski et al., 2014) measure internal and external expressions of consent for consensual sexual experiences, respectively, with the former focusing on internal feelings associated with sexual consent and the latter on behavioral expressions of sexual consent. However, both scales measure communication/interpretation of sexual consent rather than an understanding of sexual consent. Similarly, the Sexual Consent Scale-Revised (SCS-R; Humphreys & Brousseau, 2010) measures attitudes, beliefs, and behaviors regarding sexual consent negotiation between individuals but, again, does not seek to identify how individuals conceptualize sexual consent. This scale does not ask individuals to verbalize what sexual consent means to them or what sexual consent means in terms of their own behavior. Although useful to measure how individuals negotiate sexual consent and their attitudes and beliefs about sexual consent, it does not provide much insight toward identifying how individuals define sexual consent.

Qualitative research on sexual consent measurement has not been widely conducted (Peterson & Muehlenhard, 2007). For example, Jozkowski (2011, p. 113) conducted research to encourage college students to present their own definitions of sexual consent by asking them “what do you think of when you hear the words *sexual consent*?” Participants’ answers ranged from “when sex is mutually conducted between willing people” to “consent means that sex is not rape or a way to avoid rape charges.” To this extent, Jozkowski’s (2011) research provided a solid starting point for how we might measure university students’ conceptualization and definition of sexual consent. However, no further research was conducted on participants’ answers, leaving somewhat of a void between other variables (e.g., RMA, victimization history, gender) that may influence sexual consent definitions. More recently, Bednarchik et al. (2022) explored 391 undergraduate students’ sexual consent definitions and thematic analysis generated five themes: permission, agreement, willingness, wantedness, and contextual elements. These findings align with previous literature but also highlight the variety in students’ definitions.

Given that sexual consent has been inconsistently defined, if at all, in research (Beres, 2014) and the continued use of implicit sexual consent definitions suggest it is often assumed that students know what consent is, the aim of the current review is to (1) identify a consistent definition of sexual consent within the research literature focused on USEs within higher education (HE) and (2) identify a reliable and consistent method of measuring sexual consent comprehension. This review will also examine whether studies have considered contextual factors (e.g., RMA) that may impact the definition of sexual consent and/or its measurement.

## Method

This review was conducted to identify literature that could assist with answering the question: “How has sexual consent comprehension been measured when examining unwanted sexual experiences at university?” Text words were used to search nine electronic databases from January 2000 until January 2020. A manual search of internet search engines (i.e., Google Scholar) was also performed for research relating to sexual consent and university students. All searches were limited to articles published in the last 20 years and in the English language. These searches were rerun in January 2021 using the same parameters as above except the time frame was extended to January 2021. The searched databases were:

- Cumulative Index to Nursing and Allied Health Literature, Medline (Ovid), PsycINFO, Web of Science, PubMed Central (PMC)
- Applied Social Sciences Index and Abstracts, EBSCO, SAGE, Springer

An example search used for PMC included the search terms (“sexual consent” [all fields]) AND (universit* OR college* OR tertiary education OR “institute of higher learning” OR student*) [all fields]), limited to (English and 2000–2020). If advanced search options were not available, a key word search was also performed.

### Study Eligibility

#### Inclusion criteria

Studies were included if participants were aged 18 years and older and the USE measurement included within the study focused on USE that occurred while students were at university. Studies were also considered for inclusion if the study title and/or abstract included the phrase “sexual consent” (or a related phrase). Studies of experimental, cross-sectional, or intervention-based design were also included.

#### Exclusion criteria

Studies were excluded if samples included participants younger than 18 years old or not students at the time of the measured USE. Studies published outside the 20-year publication period and in a language other than English were also excluded. Studies were excluded if they were dissertations or theses.

**Figure 1.**
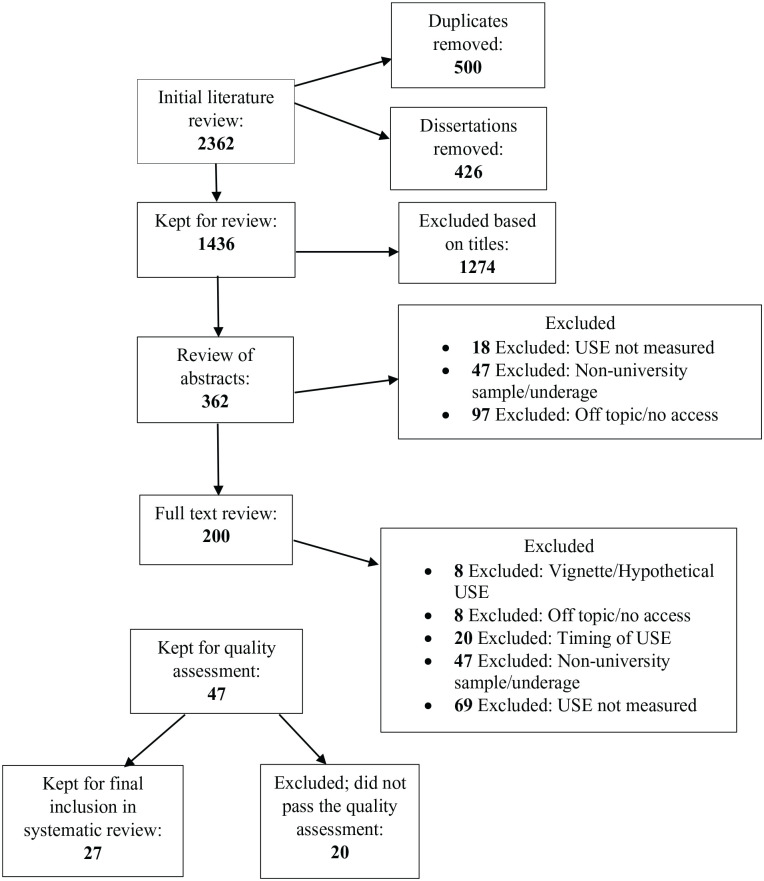
Flowchart of study selection for first review.

Two reviewers agreed on all search terms. The first literature search was conducted by reviewer one. Using the search terms stated above, the initial literature search produced 2,362 articles. Figure 1 provides an overview of the study selection process. Article references and abstracts were transferred to an electronic spreadsheet program (Microsoft Excel), and 500 duplicates were removed. Article titles were reviewed, and a further 1,074 articles were removed based on unsuitable titles with the remaining titles retained for abstract review. In total, 426 unpublished dissertations were also removed. The remaining articles were then examined for eligibility against the inclusion criteria: 162 articles were removed after abstract screening; 153 articles were removed after full-text screening. This left 47 articles eligible for quality assessment using the eight Critical Appraisal Skills Programme (2014) to assess the quality, validity, and reliability of studies. Prior to the quality assessment process, a second reviewer would quality assess every third article to reduce the risk of selection bias. The two reviewers met and discussed each quality assessment. After this discussion, 20 articles were removed leaving 27 articles for the systematic literature review. The updated literature search was conducted using the exact same process as the initial search and an additional three articles were added to the systematic review.

The data extracted from these studies included information about the authors, the publication date, the country studied, the sample size and composition, the study design, the study’s definition of sexual consent and measurement, the type of USE and its measurement, the type of analysis conducted, and the main findings of the study. These features were chosen due to lack of research surrounding definitions of sexual consent and USEs and standardized measurement tools. It was difficult to generalize findings across the studies as they varied in study design and statistical analyses. A narrative synthesis was conducted to collate the information and answer the research questions listed above.

## Results

The final review included 27 studies; an additional 3 studies were added once the searches were rerun in 2021 and 3 more were added in 2022 (see Supplemental Table 1). Most studies were cross-sectional in design (*n* = 23), were conducted at U.S. universities (*n* = 21), and included mixed gender samples (*n* = 19) (see Table 1 for these study characteristics). The total number of participants from all studies included in this review amounted to 187,013^3^ (with cisgender women accounting for approximately 63%^4^).

**Table 1.** Summary of Demographic Information by Study.

ID	Country	Gender of Sample	Sample Size	Study Focus	Consent Measure	Study Design
1	United States	F = 630, M = 351	981	V	Yes	CS
2	United States	F = 14, M = 4	18	V	Within USE	QI
3	United States	F = 55,012, M = 27,322, TG = 204	82,538	V	Within USE	CS
4	Global	F = 9,972, M = 3,905	13,877	P	Within USE	CS
5	United States	F = 3,215, M = 3,038	6,253	V	Within USE	CS
6	United States	M = 25	25	P	Within measures	LS
7	United States	M = 743	743	P	Within USE	RCT
8	Hong Kong	F = 594, M = 418	1,015	V	Within USE	CS
9	Canada	M = 10	10	P	Within USE	QI
10	Kosovo	F = 345, M = 355	700	P	Within USE	CS
11	United States	F = 3,951	3,951	V	Within USE	CS
12	Russia	F = 182, M = 156	338	V	Within USE	CS
13	United States	F = 314, M = 200, SM = 22	536	V	Yes	CS
14	United States	F = 23,980	23,980	V	Within USE	CS
15	United States	M = 326	326	P	Within USE	CS
16	Chile	F = 988, M = 322	1,310	V	Within USE	CS
17	Chile and Turkey	Chile T1: F = 832, M = 266), Turkey T1: F = 532, M = 353, Chile T2: F = 323, M = 81, Turkey T2: F = 170, M = 98	Chile T1: 1,098, Turkey T1: 885, Chile T2: 404, Turkey T2: 268	V	Within USE	LS
18	United States	F = 873	873	V	Within USE	CS
19	Poland	F = 214, M = 104	318	V	Within USE	LS
20	United States	F = 921, M = 632, SM = 26	1,671	P	Yes	CS
21	United States	CSV = 201, NO CSV = 203	404	V	Unclear	CS
22	United States	F = 5,446	5,446	V	Within USE	CS
23	United States	F = 339	339	V	Within USE	CS
24	United States	F = 4,358, M = 2,057, SM = 75	6,548	P	Within USE	CS
25	Canada	F = 88	88	V	Within USE	CS
26	United States	M = 184	184	P	Within USE	CS
27	United States	M = 217	217	P	Yes	CS
28	United States	M = 242	242	P	Within USE	LS
29	Global	F = 2,048, M = 573, GNC = 9	2,630	V	Within USE	LS
30	United States	F = 108, M = 76, GNC = 5	189	V	Within USE	MM
31	United States	Study 1: F = 550, M = 224, T/NB = 26, Study 2: F = 1,118, M = 354, T/NB = 31	Study 1: 1,067, Study 2: 1,506	V	Yes	CS
32	United States	205 (gender breakdown unclear)	205	V	Yes	MM
33	United States	205 (gender breakdown unclear)	205	V	Yes	MM

*Note.* F = female; M = male; TG = transgender; SM = sexual minority; CSV = campus sexual violence; GNC = gender nonconforming; V = victimization; P = perpetration; CS = cross-sectional; QI = qualitative interview; LS = longitudinal survey; RCT = randomized-control trial; MM = mixed-methods.

### Defining Sexual Consent

Three studies (Studies 31, 32, and 33) defined sexual consent explicitly. In Study 31, sexual consent was defined as “the freely given verbal or nonverbal communication of a feeling of willingness to engage in sexual activity.” (Humphreys, 2020, p. 184) in the preamble of the sexual consent measurement (SCS-R), sexual consent was not explicitly defined in the article. While Studies 32 (“one’s freely given verbal or nonverbal communication of their sober and conscious feelings of willingness to engage in a particular sexual behavior with a particular person within a particular context”; Marcantonio et al., 2022, p. 274) and 33 (“one’s voluntary, sober, and conscious willingness to engage in a particular sexual behavior with a particular person within a particular context”; Willis & Jozkowski, 2019, p. 1723) explicitly define sexual consent within their respective articles, it is not clear whether participants also received these definitions.

With the exception of Study 21, the remaining studies defined sexual consent implicitly and typically within the context of USE measurements (e.g., “My partner forced . . . me to have oral or anal sex”; Straus et al., 1996, p. 312). By this, we mean that they did not state the study’s operational definition of consent used throughout the study. No study used a self-report measure to define sexual consent explicitly; however, Study 7 asked participants a series of questions regarding sexual consent knowledge; Study 2 asked participants to define sexual violence (resulting in their implicit definitions of consent) and Study 31 asked participants to rate their agreement with a series of statements examining their sexual consent knowledge.

### Within Context of a Preexisting Relationship

Five studies (Studies 4, 7, 8, 10, and 12) used some iteration of the Conflict Tactics Scale 2 (CTS2; Straus et al., 1996). In this measure, sexual consent is implicitly defined in the context of a USE between intimate partners (e.g., “I used force (like hitting, holding down, or using a weapon) to make my partner have sex.”; Straus et al., 1996, p.311). To assess sexual violence, all above studies used the Sexual Coercion subscale of the CTS2 which implicitly relates sexual non-consent to three categories: use of force (e.g., “made my partner have sex without a condom”; Straus et al., 1996, p. 311, or “used force”; Straus et al., 1996, p. 312), lack of wantedness (e.g., “insisted on sex when my partner did not want to”; Straus et al., 1996, p. 312); or use of threats (e.g., “used threats to make my partner have sex”; Straus et al., 1996, p. 312).

Two studies (Studies 9 and 20) used an iteration of the Sexual Experiences Survey (SES; Koss et al., 2007) to implicitly define sexual consent within the context of a preexisting relationship. Study 9 implicitly defined sexual consent within the context of a preexisting relationship but did not implicitly (or explicitly) define sexual consent further (“how many times each act . . . occurred without consent”; Jeffrey & Barata, 2018, p. 4). Study 20 expanded this implicit definition of sexual consent to include preexisting relationships, but the definition was still within the context of an established relationship (“friend, acquaintance, casual sex partner, former sex partner, intimate partner”; Walsh et al., 2019a, p.10).

### Irrespective of Preexisting Relationship

For the three studies that explicitly defined sexual consent (Studies 31, 32, and 33), none of them featured mention of a relationship, preexisting or otherwise. Study 31 used the sexual consent definition provided by the SCS-R (Humphreys & Brousseau, 2010). Here, consent refers to “a feeling of willingness to engage in sexual activity” that must be “freely given” (Humphreys, 2020, p. 184) highlighting that lack of willingness is nonconsensual and implying that use of force and/or threat would be considered violations of consent. Studies 32 and 33 used similar definitions with slight differences. In Study 32, consent refers to “one’s . . . sober and conscious feelings of willingness to engage in a particular sexual behavior with a particular person within a particular context”; it must also be “freely given” (Marcantonio et al., 2022, p. 274). In Study 33, consent is defined as “one’s voluntary, sober and conscious willingness to engage in a particular sexual behavior with a particular person within a particular context” (Willis & Jozkowski, 2019, p. 1723). Both these studies highlight lack of willingness and incapacitation as factors concerning non-consent and implicitly consider use of force and/or threat and indicators of non-consent.

Eight studies (Studies 3, 13, 16, 17, 19, 21, 29, and 30) implicitly defined sexual consent within the context of a preexisting or unestablished relationship. Study 3 only explicitly referred to sexual consent in the context of an unestablished relationship (e.g., “were you sexually touched without your consent?”; Griner et al., 2017, p. 8), whereas more implicit phrasing was used for the intimate partner sexual violence (“have you been in an intimate [coupled/partnered] relationship that was sexually abusive—for example, forced to have sex when you didn’t want it, forced to perform, or have an unwanted sexual act performed on you?”; Griner et al., 2017, p. 7). Studies 16, 17, and 19 used the Sexual Aggression and Victimization Scale (SAV-S; Krahé & Berger, 2013) to define sexual consent, implicitly (“have sexual contact with you against his/her will by threatening to use force or by harming him/her?”; Krahé & Berger, 2013, p. 404), within the context of intimate partner sexual violence (“current or former partner”; Krahé & Berger, 2013, p. 404), acquaintance sexual violence (“friend or acquaintance”; Krahé & Berger, 2013, p. 404), and stranger sexual violence (“stranger”; Krahé & Berger, 2013, p. 404).

The SAV-S utilizes an implicit definition of sexual consent that implies both use of force and use of threats (“against his/her will by threatening to use force or by harming him/her”; Tomaszewska & Krahé, 2018, p. 7) but sexual consent conceptualization was not limited to the context of a preexisting relationship. Study 21 also implicitly defined sexual consent within and outside the context of a preexisting relationship but, notably, included no reference or definition of sexual consent; participants were asked whether they had experienced sexual assault. Study 21 implicitly referred to use of force, but explicit information related to the items was not provided. Study 13 defined sexual consent, implicitly, outside the context of a preexisting relationship such that it differentiated between dating violence (emotional and physical violence with no explicit sexual component) and sexual violence. Study 18 defined sexual consent, implicitly, outside the context of a relationship, preexisting or otherwise (“sexual activities that you did not want”; Laughon et al., 2008, p. 505). Study 29 implicitly defined sexual consent as “nonconsensual/unwanted” (Pedersen et al., 2019, p. 11) with the sexual act able to be committed by anyone, irrespective of an established relationship or gender. Conversely, Study 30 implicitly defined sexual consent as “uninvited or unwanted” (Johnson et al., 2020, p. 7) and “did someone ever use force or threat of force” (Johnson et al., 2020, p. 8).

Ten studies (Studies 1, 6, 12, 23, 25, 26, 27, 28, 29, and 34) used some iteration of the SES which does not include an explicit definition of sexual consent, rather an implied definition of sexual consent that the sexual act was “unwanted” (Koss et al., 2007, p. 1). Within the questionnaire, implicit references are made to indicate that sexual acts occurring as a result of use of force (e.g., “using force”; Koss et al., 2007, p. 2); lack of wantedness (e.g., “continually verbally pressuring me after I said I didn’t want to”; Koss et al., 2007, p. 2); and/or use of threats (e.g., “threatening to physically harm me”; Koss et al., 2007, p. 2) are nonconsensual. The only exception was Study 34 that compared the results from participants who completed an iteration of the SES and the Revised Sexual Coercion Inventory (SCI-R; French et al., 2017); this study used a version of the SES that refers to behaviors that occur “without . . . consent” (Marcantonio et al., 2022, p.4) and modified the SCI-R to also reflect this. The SCI-R (French et al., 2020, p.178) implicitly defines consent as sexual acts that occur in the absence of force (“used physical force”), threatened force (“threatened . . . physical force”), incapacitation (“encouraged me to drink and then took advantage”), and a range of coercive tactics (e.g., “begged me and would not stop”, “said things to make me feel guilty”). A summary of the results of sexual consent conceptualizations is visualized in Table 2.

**Table 2.** Summary of Sexual Consent Conceptualizations by Study.

Study	Implicit Definition	Discrete Consent Measure	Use of Force	Use of Threat	Lack of Wantedness	Incapacitated	Preexisting Relationship	Irrespective of Relationship	Lack of Willingness
1	x	TRSS	x	x	x	x			
2	x	Self-report	x		x				
3	x		x		x			x	
4	x		x	x	x		x		
5	x			x		x			
6	x		x	x	x	x			
7	x		x	x	x		x		
8	x		x	x	x		x		
9	x		x	x	x	x	x		
10	x		x	x	x		x		
11	x		x		x				
12	x		x	x	x	x	x		
13	x	IRMA	x	x		x		x	
14	x		x	x		x			
15	x		x	x					
16	x		x	x		x		x	
17	x		x	x		x		x	
18	x		x		x			x	
19	x		x	x		x		x	
20	x	SCS-R	x	x	x	x	x		
21			x		x			x	
22	x				x				
23	x		x	x	x	x			
24	x		x					x	
25	x		x	x	x	x			
26	x		x	x	x	x			
27	x	CCC	x	x	x	x			
28	x		x	x	x	x			
29	x		x	x	x	x		x	
30	x		x	x	x	x		x	
31		SCS-R	Implied	Implied				x	x
32			Implied	Implied		x		x	x
33			Implied	Implied		x		x	x

*Note.* TRSS = token resistance to sex scale; IRMA = illinois rape myth acceptance; SCS-R = sexual consent scale revised; CCC = comprehension of sexual consent/coercion scale.

### Measuring Sexual Consent

Eight studies (Studies 1, 2, 13, 20, 27, 31, 32, and 33) included a measure of sexual consent and this is further visualized in Table 2. With the exception of Study 2, all these studies used a Likert-type scale to measure participants’ (dis)agreement with the items. Study 1 used the Token Resistance to Sex Scale (TRSS; Osman, 1998) to measure the extent to which participants endorsed the belief that women use token resistance (i.e., saying no when they intend to consent to sex) as a method of consenting to sexual activity. Study 2 asked participants how they knew when a sexual situation transitioned from wanted to unwanted and the signals used to determine whether their partner had overstepped sexual boundaries. Study 13 used five statements from the Illinois Rape Myth Acceptance Scale (Lonsway & Fitzgerald, 1995) to assess participants’ understanding of, and attitudes to gaining sexual consent. Study 20 included 5 items from the Indirect Behavioural Approaches to Consent subscale of the SCS-R (Humphreys & Brousseau, 2010) to measure how participants use verbal sexual consent behaviors. Study 27 used the Comprehension of Sexual Consent/Coercion Scale (Gibson & Humphrey, 1993) to measure the extent to which participants perceive sexually coercive behavior as an appropriate method of gaining sexual consent. Study 31 included seven statements from the SCS-R (Humphreys & Brousseau, 2010) to measure participants’ knowledge of sexual consent. Studies 32 and 33 asked participants to rate the extent to which they considered their most recent sexual behaviors consensual and, if consensual, to explain their reasoning for the rating.

### Awareness or Comprehension of Sexual Consent

Eight studies (Studies 1, 2, 6, 7, 13, 19, 20, 27, 31, 32, and 33) included measures that were designed to assess participants’ awareness or comprehension of sexual consent. Using the TRSS (Osman, 1998), Study 1 found that White male participants who were part of Greek-life culture had significantly higher token resistance scores when compared to other groups in the study (i.e., non-Greek members, female Greek members). Using face-to-face interviews, Study 2 asked urban commuter university students how they knew when their sexual partner (prospective or current) “crossed the line” (Delle et al., 2019, p. 141; overstepping sexual boundaries) or when a sexual situation transitioned from wanted to unwanted. Results indicated that participants generally relied on feelings to detect this change, and some discussed the importance of establishing clear sexual boundaries. Other participants mentioned that this discussion should take place prior to the sexual event; however, one participant described sexual consent as a continuous discussion over time rather than a discrete process. Study 6 included a scenario designed to measure participants’ ability to identify sexual aggression using a scale ranging from *consensual sex* (1) to *rape* (10); the ability to identify the scenario as rape significantly increased 2 months after completing the bystander intervention program. Study 7 evaluated the efficacy of a bystander intervention program; participants either completed the bystander intervention program (intervention group) or were enrolled in a health promotion program (placebo group). The intervention program included three measures associated with sexual consent. The first was designed to identify participants’ legal knowledge (in the respective state) of sexual assault and rape. The second identified participants’ knowledge of effective consent for sex. And the third identified participants’ opinion on expected outcomes of engaging in nonconsensual sex. Significant increases across all three variables were found when participants in the intervention group were compared to the placebo group.

Study 13 surveyed university students on the efficacy of bystander interventions. The findings indicated that sexual minority students had a more accurate understanding of sexual consent (i.e., lower RMA) than heterosexual students; similarly, compared to participants who had not experienced sexual victimization, those who had experienced sexual victimization also had more accurate understanding of sexual consent. Study 19 measured the association between attitudes to sexual coercion and perpetration of sexual aggression and found a significantly positive relationship, indicating that positive attitudes toward sexual coercion were linked to perpetration. Study 20 included a single item measure of ambiguous sexual consent to attempt to reduce the risk that social desirability would prevent participants from disclosing perpetration. The researchers also included a measure of nonverbal sexual consent communication. Overall, 9% endorsed the ambiguous consent item and 26.5% of participants reporting perpetration also endorsed this item. Statistically significant correlates of ambiguous sexual consent endorsement included higher RMA and greater belief in nonverbal sexual consent practices. Study 27 surveyed college men to examine the connection between sexual comprehension and perpetration of sexual violence. The findings suggested that greater comprehension of sexual consent predicted less sexual perpetration. Furthermore, all the risk factors measured within this study (RMA; conformity to masculine norms; and peer support of abuse) were fully mediated by comprehension of sexual consent with higher RMA, greater conformity to masculine norms and greater peer support of abuse predicting less comprehension of sexual consent.

Across two separate studies, Study 31 examined the correlates of bystander intentions (intention to help) and the correlates of self-reported bystander behavior (actual bystander interventions) in the context of SV. Using a four-point Likert-type scale (*strongly disagree* [0] to *strongly agree* [4]), participants rated their (dis)agreement with seven statements from the SCS-R (Humphreys & Brousseau, 2010) designed to assess their participants’ knowledge of sexual consent. In both studies, sexual consent knowledge was significantly (and positively) correlated and associated with intent to help and self-reported bystander behavior.

Using the same dataset, Studies 32 and 33 assessed participants’ daily sexual consent perceptions and sexual behaviors over the course of 30 days. To measure sexual consent perceptions, participants responded to the question “Were these sexual acts that happened in the past 24 hours consensual?” (Willis & Jozkowski, 2019, p. 1727) using a seven-point Likert-type scale (*definitely not* [1] to *definitely* [7]). Participants were grouped into either those who rated all their sexual experiences as either “definitely consensual” or “consensual” or, those who reported at least one nonconsensual experience (e.g., responded with “definitely not consensual” or similar). Using an open textbox, participants who rated their sexual experience as consensual were then asked to describe “what was said, done, or felt to make you give this rating for consent?” (Willis & Jozkowski, 2019, p.1727). Study 32 focused on whether drinking patterns were associated with participants’ perception and communication of sexual consent. Results indicated that there were no statistically significant gender differences in sexual consent perception or communication. Concerning whether drinking patterns were related to sexual consent perceptions, participants who drank alcohol more frequently had significantly lower odds of classifying a sexual experience as either nonconsensual or questionably consensual. Similarly, those who engaged in binge drinking reported fewer questionably consensual or nonconsensual sexual experiences than those who did not binge drink: 17.6% of participants who reported binge drinking versus 40% who did not. No statistically significant differences were identified between sexual consent communication styles and drinking patterns (typical alcohol consumption and binge drinking behavior). Study 33 examined the relationship between sexual precedent (sexual history with a specific partner) and sexual consent communication between partners. First, however, Study 33 conducted a thematic analysis on participants’ responses to salient indicators of sexual consent communication between their partners. These responses were then coded as either “consent communicated” (verbal/nonverbal/explicit/implicit consent communication) or “tacit knowledge” (contextual assumptions amounting to consent). Out of 98 relationships, 15 exclusively reported communication cues compared to 7 that exclusively reported the use of tacit knowledge. The actual responses are not discussed in detail, but example responses include “I just kind of did it because she seemed ok with it,” “the eye contact said it all,” and “It just happened” (Willis & Jozkowski, 2019, p. 1729). Regarding the experiences themselves, most experiences were reported as consensual (*M* = 6.69, *SD* = 0.44) and consent was communicated in some manner (rather than assumed) 63% of the time. Lastly, participants who reported engaging in less than 575 sexual behaviors (*n* = 71) indicated less sexual consent communication as their sexual activity increased; for those reporting more than 575 sexual behaviors (*n* = 27), sexual communication increased.

## Discussion

This review sought to identify a consistent definition of sexual consent within the research literature focused on USEs within HE and identify a reliable and consistent method of measuring sexual consent. The current review examined the content of 33 studies on both these areas (i.e., sexual consent definition and sexual consent measurement). Additionally, studies were examined for the inclusion of contextual factors that might influence or relate to sexual consent (e.g., RMA).

### Sexual Consent Definitions

Sexual consent is at the root of defining all sexual crimes. It would seem a foregone conclusion that all research in this area would be explicit and consistent (where possible) regarding sexual consent definitions. However, only three of the studies in this review included an explicit definition of sexual consent (Studies 31, 32, and 33)—within this group, only Study 31 indicated that the participants received this sexual consent definition. The remaining studies varied on how each study implicitly conceptualized sexual consent. Nine studies included a type of sexual consent measurement, but it is not clear whether these measures were assessing the same concept (namely, sexual consent). For example, Study 13 used 5 items from an RMA measure to measure certain behaviors or attitudes associated with sexual consent. Though the relationship between RMA and sexual consent comprehension has been identified within this review (Study 27) and outside this review (Kilimnik & Humphreys, 2018; Peterson & Muehlenhard, 2004), it is not clear whether the relationship between these two constructs is sufficiently interdependent to use these measures interchangeably.

#### Explicit definitions

Study 31 and Studies 32 and 33 varied in both their explicit definition of sexual consent and with whom this definition was shared. All three studies referred to sexual consent as a type of “willingness”; for Studies 31 and 32, this was a feeling of willingness. The feeling of willingness typically refers to the mental act (or, internal state) that relates to a person’s decision to consent (Beres, 2007; Hickman & Muehlenhard, 1999; Muehlenhard et al., 2016). Relying solely on feelings to judge whether a person has consented is problematic because these feelings are not externalized (this would be an expression of willingness rather than a feeling), therefore, any other actors in the sexual situation would be unaware of the other person’s internal state; within policy and law, it is far more likely that sexual consent would be conceptualized as observable behavior. Given that both Studies 32 and 33 sought to report students’ sexual consent perceptions of their own experiences, it seems puzzling that it appears that they did not provide participants with this particular (or any) sexual consent definition. In both studies, participants were free to comment on their partner’s cues, their own cues, or contextual cues indicating consent: by asking participants to consider willingness and consent, results may have enriched our understanding of how students understand that specific nuance but, instead, we are still left with gaps to fill.

All three studies also referred to sexual consent as something that is “freely given” (or voluntary); though not explicitly stated, this would suggest that an act would not be consensual if it involved threats, force, or manipulation. However much like it is not clear whether students understand consent in the same way that researchers understand it, they also may not fully understand the implicit connotations of “freely given” agreement. Students may not fully understand coercion in this context (Palermo et al., 2022; Thomas et al., 2017): some may normalize coercive behavior and view it as part of the dating process (Burkett & Hamilton, 2012; Romero-Sánchez & Megías, 2015); others may not view coerced sexual experiences as related to sexual violence because there is no physical violence inflicted (Cleere & Lynn, 2013; Jeffrey & Barata, 2017).

Studies 32 and 33 both highlighted sobriety and consciousness as important factors related to sexual consent, neither factor were included in Study 31’s definition. This is surprising because the SCS-R was developed using university students (Humphreys & Brousseau, 2010) where overconsumption of alcohol is a common occurrence and because there seems to be confusion amongst students regarding alcohol and consent (Hirsch et al., 2019; Marcantonio & Jozkowski, 2021; Marg, 2020). As such, it would seem important to clarify to any participants (particularly those who are students) that any person who is heavily intoxicated (or unconscious) may not be capable of consenting.

#### Implicit definitions

Of the definitions that were implied, or referred to, within measures, four themes emerged: incapacitation; use of force; use of threats; and lack of wantedness. The first three themes are commonly present in the legal definition of sexual consent (Breiding et al., 2016; Dowds, 2020) and indicate that the individual’s agreement (or, consent) was obtained under duress. Lack of wantedness, however, presents an interesting dilemma: does one have to want sexual contact to consent to it? That wantedness is inherent in defining sexual consent may be linked to research that has identified participants’ preoccupation with using some form of intuition to ascertain whether their sexual partner is consenting (Bednarchik et al., 2022; Hirsch et al., 2019; Jozkowski & Peterson, 2013; Marg, 2020). This was further present in how Study 2’s participants discussed sexual consent (albeit, implicitly).

Research has begun to explore the role of wantedness in relation to unacknowledged nonconsensual sex (Hills et al., 2020; Peterson & Muehlenhard, 2007), unwanted but consensual sex (Bay-Cheng & Eliseo-Arras, 2008), and sexual regret (Johnson et al., 2020). Results seem to indicate that wantedness plays a role in how individuals conceptualize consensual sex, such that lack of wantedness is more indicative of a USE than a consensual sexual experience (Hills et al., 2020; Peterson & Muehlenhard, 2007). Despite the lack of attention given to wantedness in the legal definition, research indicates that wantedness (or lack thereof), or perceived sexual consent, may impact the outcome of USEs for some individuals (Artime & Peterson, 2015; Kern & Peterson, 2018; Peterson & Muehlenhard, 2004).

Seven studies focused on intimate partner violence; thus, sexual consent was implicitly defined within the context of a preexisting relationship. In contrast, seven studies defined sexual consent within the context of a preexisting relationship and outside of this context. Emphasizing, implicitly or otherwise, a differentiation between sexual consent negotiations in preexisting couplings versus unestablished couplings, as in Study 13, runs the risk of suggesting that sexual consent is not necessary in established relationships. Unfortunately, research seems to suggest that university students may believe this already (Hirsch et al., 2019; Marg, 2020). Historically, this harkens to an age where men could not be accused of raping their wives (i.e., marital rape) because sex between a husband and wife was, by definition, consensual (Bergen, 2006). A tendency to believe that this is still true can often indicate that an individual is more accepting of rape myths (Ferro et al., 2008). Broadly speaking, this is problematic because it implies that there are situations where sexual consent can be assumed without verbal or nonverbal indicators and suggests that prior, or current, sexual history is a relevant indicator of sexual consent.

Three studies remarked on the lack of clear definitions of sexual consent in research nevertheless did not explicitly define it (Studies 8, 21, and 34). Only two studies included in this review, outside of implied definitions within the text, identified how the researchers themselves defined sexual consent (Studies 32 and 33) but even in these studies, it is not clear whether participants received these definitions. Furthermore, Study 13 (Mennicke et al., 2019, p. 11) used wording in one question on their sexual victimization measure (“has someone had contact with you involving penetration or oral sex without your active, ongoing voluntary agreement?”) that amounted to sexual consent yet, rather puzzlingly, used three additional types of phrasing in the preceding questions to refer to sexual consent. If it cannot be assumed that implicit references of sexual consent are consistent within measures, how can we rely on consistency outside studies? Furthermore, research regarding gender differences in item wording suggest that male participants may conflate wantedness with sexual consent, such that if sexual consent is not explicitly stated, they assume that the experience is consensual (Rueff & Gross, 2017). Therefore, if they were presented with different item wording, the data may incompletely capture responses.

### Rape Myth Acceptance

Little is known about the exact relationship between sexual consent comprehension and RMA within the HE research context, yet it seems likely that there is a relationship because those high in RMA are more likely to possess less sexual knowledge (Aronowitz et al., 2012), hold traditional gender beliefs that excuse sexual aggression (Adams-Curtis & Forbes, 2004), and be more accepting of sexual coercion (Warren et al., 2015). Two studies (Studies 6 and 7) measured RMA but, surprisingly, did not examine the relationships between RMA, legal knowledge, sexual consent comprehension, and sexual violence perpetration. Presumably, improving legal knowledge would result in an improvement in sexual consent comprehension, thus, leading to a reduction in sexual violence perpetration. Research of police officers (who would, presumably, have sound legal knowledge; Sleath & Bull, 2015; Venema, 2018) and law students (Krahé et al., 2008; Sleath & Bull, 2015) has suggested that RMA supersedes legal knowledge, so, perhaps prevention also requires a reduction in RMA to be effective long term. Inarguably, there is some relationship between sexual consent knowledge or, perhaps, attitudes to sexual consent negotiation that is linked to RMA (Warren et al., 2015; Yapp & Quayle, 2018). However, RMA measures should not replace measures (or research) that seek to understand how students define sexual consent. The lack of research in this area suggests that a firmer understanding is required as to how students define and comprehend sexual consent before isolating factors that contribute to this.

### Sexual Consent Measurements

The measurements included in this review, overall, measure certain attitudes and behaviors related to sexual consent communication (e.g., Humphreys & Brousseau, 2010) or that may predict sexually aggressive behavior (e.g., Tharp et al., 2013). Study 31 modified the SCS-R (Humphreys & Brousseau, 2010) to assess knowledge rather than attitudes and beliefs about sexual consent. Though the authors note the number of statements included in their modified version (seven) and justify this change to ensure that knowledge is assessed, it is unclear which statements were removed. Considering the original SCS-R includes 39 statements, this makes it difficult to identify how consent knowledge was actually assessed. Their modified SCS-R uses a four-point Likert-type scale, and the original version uses a seven-point Likert-type scale yet there is no discussion for the rationale of this modification.

Study 33 came the closest to measuring students’ cognitive conceptualization of sexual consent—namely, the words they would use to describe or explain it. By exploring consent in this way, we can view it more as an expression of willingness (Beres, 2007; Hickman & Muehlenhard, 1999; Muehlenhard et al., 2016) rather than simply a latent construct measured by Likert-type scales assessing attitudes and beliefs. In Study 33, participants’ actual responses regarding indicators of sexual consent are not discussed in detail, but the authors note that these responses may not represent best practice in consent communication. Furthermore, despite acknowledging that some responses suggest that a partner has assumed the other’s consent, there is no discussion about the differences between sexual consent perception and responses. This would seem important because some responses might suggest questionably nonconsensual/nonconsensual sexual experiences rather than consensual. For example, one participant stated, “I just kind of did it because she seemed ok with it” (Willis & Jozkowski, 2019, p. 1729) which could be considered questionably consensual (or nonconsensual) because it is not clear whether consent was established prior to the act or, how their partner consented to the act. As their research suggests, consent communication may change over the course of a relationship, but this does not mean it is any less important to ensure that sexual experiences are consensual.

Similarly, Study 2 encouraged students to explain how they recognized and defined sexual violence with violations of sexual consent referred to as “crossing the line” (Delle et al., 2019, p.141). Students reported the use of a “gut feeling” (Delle et al., 2019, p. 141) to determine when a sexual experience evolved from wanted to unwanted and/or nonconsensual. Here, students unknowingly revealed their implicit understanding of sexual consent. Other students discussed how certain actions (e.g., paying for dinner) represent implicit cues indicating the occurrence of a sexual encounter (e.g., accepting dinner is an implicit agreement to engage in a sexual act). Unfortunately, sexual consent knowledge was not the primary focus and not further explored. The results of this study stress the importance of identifying how students individually and implicitly define sexual consent as it may inform how they define (and recognize) sexual violence.

Considering students’ individual sexual consent definitions could develop this research field in several ways. First, the relationship between rape myths (or other stereotypical beliefs and attitudes) and sexual consent conceptualization could be better understood. Haugen et al. (2018), for example, have demonstrated the relationship between definitions of rape and rape myths but how sexual consent fits into this relationship has not been explored. Second, measuring students’ baseline sexual consent knowledge allows for the development of programs that target areas where students lack knowledge as opposed to programs that do not reflect the student experience. Better sexual education of university students has been consistently mentioned within this field and by advocacy organizations for universities (e.g., Universities UK, 2016), however, not tailoring these programs to be more representative of students’ thinking processes appears to be an oversight. Finally, measuring students’ initial baseline knowledge affords educators the opportunity to determine whether these programs are working or require further development. Based on the results of this review, however, there was little consensus on how sexual consent should be defined or measured in the context of USEs.

### Limitations

The current review has several limitations. Studies were excluded if there was no mention of experienced university-based USEs. For example, research involving participants discussing hypothetical scenarios or situations were excluded unless participants’ own experiences were measured. Studies were excluded if the USE measurement did not specify that the USE had to occur while at university. It is possible that some studies who did not explicitly state when the USE had occurred may have been missed. Though this review involved a mixture of qualitative and quantitative research, much of the data relied on self-report measures and no study provided details of objective measurements of USEs (e.g., police statistics, campus reports). Participants had to be 18 years or older to be included in the review to allow for greater generalizability across participants. As a result, studies including 17-year-old university students were excluded. Similarly, most of the studies were from U.S. universities and, primarily, involved Caucasian heterosexual able-bodied individuals so the generalizability of these results to more diverse populations is limited. Of the limited research available (e.g., Griner et al., 2021; Marcantonio & Willis, 2022; McKie et al., 2020; Walsh et al., 2019b), results appear to be comparable to their heterosexual peers though some differences have been identified (e.g., HIV/AIDs status, sexual role preference [Sternin et al., 2022]) and this warrants further examination. However, while similarities have been identified, rather than providing opportunity for generalizability, it instead suggests the need for further research because these similarities indicate that nonheterosexual individuals may internalize traditionally heteronormative scripts in lieu of scripts relating to their own sexual orientation. Broadly speaking, more research needs to consider the differences between sexual and gender minorities, particularly as they tend to receive less relevant sexual education (Epps et al., 2021; Hobaica & Kwon, 2017; Rabbitte, 2020).

## Conclusion

In 2007, Beres (p.105) concluded that “many scholars fail to define consent explicitly . . . [Ostler, 2003], forcing the readers to rely on assumed definitions.” This review confirms that, in the context of USE-based work, little has changed. Sexual consent can be subjective; therefore, it is unreasonable to expect university students to conform or respond to standards that do not reflect their life. For university students, sexual consent is contextual and perhaps reflective of their sexual inexperience and adaptation to the university setting. From this perspective, it seems unlikely that sexual consent could ever be completely understood using a Likert-type scale.

We must learn more from university students—their sexual consent definitions, their sexual consent operationalization and how they communicate and discuss consent in real-life settings. In-depth methodological approaches that center on the students’ experience provide the most optimal means of doing so. Learning from students will help to shape educational interventions that increase the likelihood that students utilize their new knowledge outside the classroom. Furthermore, consolidation of research findings across studies allows for the progression of the field of study toward effective and targeted interventions, such consolidation is unachievable if researchers themselves are not explicit in their definitions. Such transparency is imperative as we move forward.

## Supplemental Material

sj-docx-1-tva-10.1177_15248380221147558 – Supplemental material for Defining and Measuring Sexual Consent within the Context of University Students’ Unwanted and Nonconsensual Sexual Experiences: A Systematic Literature Review Click here for additional data file. Supplemental material, sj-docx-1-tva-10.1177_15248380221147558 for Defining and Measuring Sexual Consent within the Context of University Students’ Unwanted and Nonconsensual Sexual Experiences: A Systematic Literature Review by Ngozi Anyadike-Danes, Megan Reynolds, Cherie Armour and Susan Lagdon in Trauma, Violence, & Abuse

sj-docx-2-tva-10.1177_15248380221147558 – Supplemental material for Defining and Measuring Sexual Consent within the Context of University Students’ Unwanted and Nonconsensual Sexual Experiences: A Systematic Literature Review Click here for additional data file. Supplemental material, sj-docx-2-tva-10.1177_15248380221147558 for Defining and Measuring Sexual Consent within the Context of University Students’ Unwanted and Nonconsensual Sexual Experiences: A Systematic Literature Review by Ngozi Anyadike-Danes, Megan Reynolds, Cherie Armour and Susan Lagdon in Trauma, Violence, & Abuse

## Notes

1. In this article, unwanted sexual experience is a collective term grouping together unwanted and nonconsensual sexual behaviors including non-penetrative, attempted penetrative, and completed penetrative sexual acts.

2. Though we predominantly refer to sexual consent, in the context of this article, we are referring to “non-consent” unless otherwise specified.

3. Studies 32 and 33 involve the same sample.

4. Studies 32 and 33 did not disaggregate their data by gender.
