# Supplementary material for: Defining and Measuring Sexual Consent within the Context of University Students’ Unwanted and Nonconsensual Sexual Experiences: A Systematic Literature Review
Source: Trauma Violence Abuse. 2023 Jan 14;25(1):231–45. doi: 10.1177/15248380221147558 (PMC10666479; doi:10.1177/15248380221147558)
Supplement: sj-docx-1-tva-10.1177_15248380221147558 – Supplemental material for Defining and Measuring Sexual Consent within the Context of University Students’ Unwanted and Nonconsensual Sexual Experiences: A Systematic Literature Review [file sj-docx-1-tva-10.1177_15248380221147558.docx]

| Supplementary Table 1.  *Summary of findings from systematic review* | | | | | | | | | | | |  |
| --- | --- | --- | --- | --- | --- | --- | --- | --- | --- | --- | --- | --- |
| Author & Date | **Country** | | **Sample size** | | **Design** | **Consent definition** | **Consent measure** | | **SV measure(s)** | | **Main findings** |  |
| 1. Canan et al. (2018) | USA | 981 (F=630, M=351) | | CS | | Implicit: coercion, threats, force, incapacitation | TRSS | SES-R | | - Gender, Greek status and race/ethnicity were predictors for endorsing token resistance  - Gender, Greek status and race/ethnicity were significant predictors of RMA - Greek men and higher token resistance and RMA than any other group - Women more frequently report experiences of rape and SA - Men report high rates of SA because refusal was useless | | |
| 1. Donne et al. (2019) | USA | 18 (F=14, M=4) | | QI | | Implied: lack of wantedness, lack of consent, force/threat | Unclear | "Have you ever had SC (kissed, touched, or done anything sexual) that you consider to be unwanted or without your consent in any way?" "Has anyone ever tried or made you have sex with them, when they knew you did not want to?" | | - Participants used their feelings to guide recognition and labelling of nonconsensual sexual behaviour - Timing of recognizing and acting on your feelings was also raised - Role of feelings in recognizing SV, not only in the moment but also afterwards - Explicit communication, establishing agreements around acceptable behaviour and the role of consent emerged for some but not all - Dynamism of sexual interactions and the role of giving consent or not to each step or aspect of it, in order to make it fully consensual and enjoyable for both parties - Consent could be "implied" - When asked how they knew that a sexual experience is "good/bad, wanted/unwanted, forced/not forced, coerced/not coerced": - Some students described "unwanted" sexual experiences as ones when "something happens and you don’t want to, but you let it happen anyway" because you "don’t know how to say no".  - Another described it as something that "you do just to make the other person happy". - Other experiences that qualified as "unwanted" included ones where the students did not feel comfortable saying no, for example in the past when they were younger, so they tried to just go through it without expressing their feelings.  - A clear definition of an "unwanted" sexual experience involved being intoxicated and/or not being aware of what is happening. | | |
| 1. Griner et al. (2017) | USA | 82538 (F=55012, M= 27322, TG=204) | | CS | | Implied: without consent, force, lack of wantedness | Included in question with SV (NCHA-II) | NCHA-II | | - 6% sexually touched without consent - 3% experienced attempted sexual penetration without consent - 1.5% were sexually penetrated without consent - 2% were in a sexually abusive relationship - TG students reported consistently higher percentages of victimization experiences than m/f - TG students (compared to males) reported sig higher odds of experiencing all 9 types of victimization - Highest odds in attempted and sexual penetration without consent - Females had higher odds of experiencing 7/9 types of victimization when compared with male students, females reported lower odds of experiencing physical assault and verbal threats | | |
| 1. Gámez-Guadix et al. (2011) | Global | 13877 (F=9972, M=3905) | | CS | | Implied: force, lack of wantedness, threats, coercion | None | Sexual coercion subscale CTS2 | | - 26.7% males and 19.6% females reported verbally coerced sex - 2.4% males and 1.8% females physically forced to have sex - Prior victimization associated increased probability of ASB - ASB associated with increased probability of verbally coercing and physically forcing sex | | |
| 1. Herbenick et al. (2019) | USA | 6253 (F=3215, M=3038) | | CS | | Implied incapacitation, lack of consent, force, or threat | Included in question with SV (modified from NCWSV) | - AOD penetrative sex  - Penetrative sex using force/threat - Oral sex using force/threat | | - 15.8% of women and 7.7% of men reported that they had experienced non-consensual penetration  - 1.2% men, 1.8% women reported that someone had oral, vaginal, or drugged them since college - 1.8% of women reported being made to have penile-vaginal intercourse through the use of force or threats to harm them or someone close to them  - 0.9% of men and 1.6% of women reported that someone forced/threatened them to have oral sex  - 6.9% men, 14.1% women reported that someone had non-consensual sex with them (AOD)  - Highest rates of non-consensual sex were reported by men identifying as gay/homosexual (15.2%) or bisexual (14.8%), 7.0% of straight  - 251 men, 549 women experienced +1 incident - Stranger/acquaintance: ~8% men, 5% women/almost 33% men, 44% women  - Friend: almost 33%  - Boyfriend/girlfriend: ~8% men, 9% women  - Missing class/de-enrolment: ~2% men, 5% women  - MH: ~7% men, 19% women | | |
| 1. Orchowski et al. (2018) | USA | 25 men | | CS | | Implicit: coercion, threats, force, incapacitation | Not measured | - IRMA (Payne et al., 1999) - SES-SFP (Koss et al., 2007) - Hypergender ideology scale (Hamburger et al., 1999) - scenario (Pinzone-Glover et al., 1998) - sexual social norms inventory (Bruner, 2002) | | - Less endorsement of rape myths at 2 months - Fewer alcohol related consequences - Less engagement in sexual coercion - Of men who perpetrated after 2 months, majority had hx of sexual aggression at baseline - Men who perpetrated were less likely to label hypothetical scene as rape | | |
| 1. Salazar (2014) | USA | 743 men | | RCT | | Implied: force, threats, coercion, lack of wantedness | Not measured explicitly | CTS2 | | - Program participants reported less SV perpetration - RMA decreased in program participants - Increase in empathy for victims - Legal knowledge increased & consent knowledge | | |
| 1. Wong (2019) | Hong Kong | 1015 (F=594, M=418) | | CS | | Implied: force, threats, coercion, lack of wantedness | Not measured explicitly | - Chinese CTS2  - Adverse childhood experience | | - Sexual minority significantly associated with IPSV; also being older, a smoker and having experienced CSA - IPSV survivors had more severe anxiety, depression symptoms, psychosomatic symptoms and poorer QoL | | |
| 1. Jeffrey & Barata (2018) | Canada | 10 men | | QI | | Implicit: coercion, threats, force, incapacitation | Not measured explicitly | SES (SFP) | | - Described violent and coercive tactics ranging from verbal pressure and persistence to physical force - Tactics were generally an attempt to acquire intercourse but were sometimes used for other activities (e.g. those they had not attempted as a couple) - Men’s SV tactics and accounts were patterned by dominant discourses about heterosexuality - Some of the men explicitly linked their use of SV their insistence or expressions of frustration following a refusal, or ignoring signs of nonconsent  - Attributed these behaviors with sex being pleasurable or not wanting or being able to stop - Others linked their expressions of frustration or ignoring signs of nonconsent to male sexual needs and expectations of finishing sex (i.e., men reaching orgasm) - Almost all participants used qualifications and “just-” statements that minimized their behaviors and distanced them from something more severe (often from physical force) - Most participants described at least some of their SV (even nonphysical tactics) as bad, wrong, or selfish, or described feeling bad, guilty, disappointed in themselves, or even abusive - Few men spoke meaningfully about what [consent] looked like in their relationship (though this may have been due, in part, to the nature of the interview questions) and many provided contradictory accounts - Some men discussed consent and mutuality but also described circumstances in which they tried to see how far they could push it (e.g., when they did not get a clear refusal) | | |
| 1. Kelmendi & Baumgartner (2017) | Kosovo | 700 F=345, M=355) | | CS | | Implied: lack of wantedness, threat, coercion, force | Not measured explicitly | CTS2 | | - Males reported higher rates of perpetration for each type of IPV - majority of acts were minor - students who had perpetrated or had been victims of physical, psychological, or SV also experienced higher levels of violence socialization in comparison with those who did not perpetrate or experience any IPV -Males who had been either perpetrators or victims of IPV (physical, psychological, and SV) showed higher rates of violence socialization in comparison with females - Those who had perpetrated or experienced any IPV had more tolerant attitudes toward violence. Males who had been either perpetrators or victims of any IPV showed higher rates of violence approval in comparison with females | | |
| 1. Krebs et al. (2011) | USA | 3951 women | | CS | | Implied: unwanted or forced sexual contact | Not measured explicitly | - Forced touching of a sexual nature  - Oral sex  - Sexual intercourse - Anal sex  - Sexual penetration with a finger or object | | - Before college: 14.9% experienced attempted or completed SA; 8.3% attempted SA, 10.5% completed SA; 8.7% physically forced SA, 3.4% incapacitated SA - During college: 14.2% experienced attempted or completed SA; 7.8% attempted, 9.6% completed; 4.8% physically forced, 6.2% incapacitated - being an undergraduate women at an HBCU is associated with significantly lower odds of experiencing any SA since entering college - HBCU women had a lower odds of experiencing incapacitated SA since entering college - HBCU women were less likely to experience incapacitated SA prior to entering college than their non-HBCU counterparts - HBCU women had higher odds of experiencing physically forced SA prior to entering college - Factors associated with race/ethnicity, rather than something unique to the HBCU environment, are responsible for the lower rates of SA found among HBCU women - Experiencing SA before coming to college is associated with an increase in the likelihood of experiencing SA during college | | |
| 1. Lysova & Douglas (2008) | Russia | 338 (F=182, M=156) | | CS | | Implied: unwanted sexual activity | Not measured explicitly | CTS2 | | -25.5% were the victims of one or more physical attacks by their partner in the previous 12 months - 31% reported perpetrating violence against their partner - 24.1% reported that their partner had used some form of coercion to have sex in the previous 12 months - 23.6% reported coercing their partner for sex - 3.1% reported receiving threats to obtain sex - 3% reported victimizations that included physical force - 5% experience the most severe coercion (threatening the partner to get sex/physically forcing sex) - 2.9% reported perpetrating severe sexual coercion - 32.8% of females vs. 15.4% of males reported being victims of sexual coercion - 24.4% of males vs. 22.9% of females reported perpetrating sexual coercion against a partner | | |
| 1. Mennicke et al. (2019) | USA | 536 (F=314, M=200, SM=22 | | CS | | Implied force, threats, coercion; incapacitation | - IRMA | SV items modified from the American Association of Universities survey (Cantor et al., 2015) | | - SM students had more accurate understandings of sexual consent and lower perceptions of institutional support and college connection - Victims of violence had more accurate understandings of sexual consent but college connection and perceptions of institutional support were equivalent between victims and non-victims - Experiencing SM based discrimination was related with lower feelings of connection to the college and perceptions of institutional support | | |
| 1. Mohler-Kuo et al. (2004) | USA | 23980 women | | CS | | Implied threats, force, and intoxication | Not measured | (1) Rape while forced  (2) Rape while threatened (3) Rape while intoxicated | | - Approximately 1 in 20 women experienced rape since the beginning of the school year - No significant differences across years except rape by threat - rape while intoxicated the major type of forced sex (72%) - School heavy episodic drinking level was the strongest risk factor for being rape whilst intoxicated - College women from medium to high level episodic drinking were 1.5-1.8x more at risk of being rape whilst intoxicated - Students in rural environments had 1.3x increased risk of being raped whilst intoxicated | | |
| 1. Richardson et al. (2017) | USA | 326 men | | CS | | Implied threats, coercion, and force | Not measured | - I got my date drunk or stoned - I threatened to terminate the relationship - I said things to make the other person feel guilty - I tried to turn date on by touching him/her even though he/she wasn't interested - I made false promises about the future of the relationship - I physically held my date down (Tyler et al., 1998) | | - 43.3% perpetrated at least one type of sexual coercion; 27.4% got their date drunk/stoned - 15.3% false promises about future of relationship - 9.3% force to SA - 73% oral/sexual intercourse resulted from coercive behaviour - Only interparental hostility and sense of entitlement were significantly correlated with perpetration of sexual coercion - Hostility between parents was found to be marginally significantly associated with perpetration of sexual coercion - Interparental warmth and hostility, inconsistent parenting, overparenting, and feelings of entitlement accounted for 10.8% of the explained variance in the perpetration of sexual coercion. - Significant indirect association found between inconsistent parenting and sexual coercion through feelings of entitlement  - There was a significant indirect effect between overparenting and sexual coercion through feelings of entitlement | | |
| 1. Schuster et al. (2016) | Chile | 1310 (F=988, M=322) | | CS | | Implicit: against your will | Not measured | SAVS | | - Last 12 months: use/threat of physical force was the most common strategy of being victimized by SV, and highest in men across all relationship classes - Last 12 months: verbal pressure most common strategy for perpetration of SV for men and women as partner, threat/force for men as friend/acquaintance and exploitation/verbal pressure for women and; threat/force for women as stranger and exploitation for men | | |
| 1. Schuster & Krahé (2019) | Chile & Turkey | Chile T1: 1098 (F=832, M=266) Turkey T1: 885 (F=532, M=353) Chile T2: 404 (F=323, M=81) Turkey T2: 268 (F=170, M=98) | | LS | | Implicit: against your will | Not measured | SAVS | | - Chile: 17.6% and 7.8% of men/women perpetrated sexual aggression in the last 12 months - Turkey: 26.6% and 21.9% of men/women perpetrated sexual aggression (difference NS) - More risky sexual scripts indirectly increased the probability of sexual aggression perpetration at T2 through more risky sexual behaviour and past perpetration in both the Chilean and Turkish sample - Higher sexual self-esteem was associated with higher initiation assertiveness and indirectly increased the probability of sexual aggression perpetration at T2 through perpetration at T1, but only in the Turkish sample - Higher religiosity indirectly decreased the probability of sexual aggression perpetration at T2 via less risky sexual scripts, less risky sexual behaviour, and a lower likelihood of perpetration at T1 in both samples - In the Turkish sample only, higher religiosity increased the likelihood of perpetration at T2 via lower sexual self-esteem and a higher probability of perpetration at T1 | | |
| 1. Sutherland et al. (2017) | USA | 873 women | | CS | | Implicit: force, lack of wantedness | Not measured | AAS | | - 22% reported that they had experienced IPV/SV during the preceding fall semester - Most college women reported that they were not asked about their experiences with IPV/SV during their most recent visit to the college health centre - Only 10.2% (n = 89) of the participants reported that they were screened for IPV/SV - College women were 4.5x more likely to report IPV/SV screening if they went to the college health centre for a gynaecological reason, nearly 2x as likely to report IPV/SV screening if they lived off-campus  - College women whose mothers had at least a baccalaureate degree were approximately half as likely to report IPV/SV screening as those with less educated mothers  - College women from public colleges/universities were 4x more likely to report IPV/SV screening compared with those from private institutions  - College women who attended urban colleges/universities were 2.5x times more likely to report IPV/SV screening than those who attended rural and suburban schools | | |
| 1. Tomaszewska & Krahe (2018) | Poland | 318 (F=214, M=104) | | LS | | Implicit: against your will | Not measured | SAVS | | - 28.6% women and 25.5% men victimized at least once in last year - 8.7% men and 7.5% women reported perpetration at least once in last year - SV since 15-last year was significant predictor of SV within the last year - Risky sexual scripts assessed at T1 indirectly predicted SV in the last year via risky sexual behaviour and past experiences of victimization - sexual self-esteem showed a negative indirect path to SV in past year via SV since 15-last year  - Lower sexual self-esteem at T1 increased the probability of being SV within last year through past SV in adolescence - Pornography use indirectly increased the odds of SV in last year via more risky sexual scripts, more risky sexual behaviour, and SV since 15-last year  - Religiosity indirectly decreased the odds of last year SV via less risky sexual scripts and behaviour and the absence of SV before T1  - Pornography use was an indirect predictor of the perpetration of sexual aggression in the last year via perpetration since 15-T1  - Attitudes toward sexual coercion were prospectively linked to the perpetration of sexual aggression within the last year - More frequent pornography use was positively linked to attitudes toward sexual coercion and to risky sexual scripts | | |
| 1. Walsh et al. (2019) | USA | 1671 (F= 921, M= 632, SM= 26) | | CS | | Implicit: coercion, threats, force, incapacitation | - ambiguous consent - use of non-verbal consent tactics (five items SCS-R (Indirect Behavioural Approaches to Consent subscale) | SES-R (P) | | - 2.1% reported perpetration since starting college, most common sexualized touching, least common completed penetration - 3.3% men and 1.4% women reported perpetration - Men reported higher prevalence of unwanted touching but an equivalent amount of completed penetration - 1.3% reported perpetration from the start of the year - Correlates of perpetration included past-year SV (OR=3.0), SA perpetration prior to college, monthly binge drinking (OR=2.3), higher depression (O =2.0), higher levels of traditional masculinity (OR=1.5), and greater belief in nonverbal consent communication (OR=2.1) - Social/interpersonal correlates included more use of nonverbal consent strategies (O =3.4) - Overall, 9% said yes to the ambiguous consent item - 26.5% reported perpetration and ambiguous consent - Correlates of ambiguous consent included older age (OR=1.4); having difficulty paying for basic necessities (OR=1.4)/(OR=2.4); identifying as non-heterosexual (OR=2.3)/(OR=2.4); experiencing CSA (OR=3.0)/prior SV (OR=2.2; more frequent use of alcohol/drugs before sex (OR=2.2); worse depression (OR=1.8), anxiety (OR=1.5), and self-esteem (OR=1.7); and higher RMA (OR=1.4) and greater belief in nonverbal consent practices (OR=1.3) - Social/interpersonal correlates included having hookups (vs. steady/exclusive relationships; OR=4.7) and, use of nonverbal consent tactics (OR=1.7) | | |
| 1. Rothman et al. (2019) | USA | 404 women (CSV=201  no CSV=203) | | CS | | Not defined | Not measured | Participants in the C-SA group provided information about the timing of the C-SA relative to the academic year of college, the assault type, the severity of the assault, their relation to the perpetrator, physical injuries resulted from this assault, reported this assault to authorities, medical treatment following the assault. | | - Women who experienced C-SA experienced significantly poorer functioning following the assault than did the control group - Women who experienced C-SA had significantly greater PTS symptoms, depression, and anxiety symptoms about 9 years post-assault - Women with a history of C-SA who endorsed being in a current relationship, compared to those in a current relationship without a C-SA history, reported significantly lower levels of emotional intimacy and sexual intimacy with their current romantic partner (approximately 9 years post-assault)  - PTS symptoms 9 years post-assault were significantly higher when the assault resulted in physical injury, more fear at the time of the assault and the assault consisted of more than one type of penetrative assault - Anxiety symptoms 9 years post-SA were significantly higher when women reported more fear at the time of their assault or experienced more than one type of penetrative assault in a single incident - Women reported significantly higher depressive symptoms 9 years after the assault when the assault consisted of more than one type of penetrative assault - Women whose SA resulted in physical injury reported significantly lower emotional intimacy with their current partner at the time of the study - Results indicated that those who were assaulted by a stranger reported no differential mental health or relationship functioning on any outcomes compared with those who were assaulted by a friend, family member, or current/past romantic partner | | |
| 1. Krebs et al. (2009) | USA | 5446 women | | CS | | Implied: nonconsensual or unwanted SC | Not explicitly measured | College SA web-based survey | | - 19% reported experiencing completed or attempted SA since entering college - Since entering college, slightly more experienced completed SA (13.7%) than attempted SA (12.6%), with 7.2% experiencing both completed SA and attempted SA during college - Nearly 5% of the total sample were forcibly SA since college entry  - More than 3% experienced forced rape and 1.4% experienced forced sexual battery since college  - AOD SA experienced by 7.8% since college  - 2.5% experienced AOD SA before/since college, 1.4% experienced forced SA before/since college - Senior year of college had greatest cumulative prevalence of each type of completed SA - ~9% surveyed in their sophomore year experienced completed SA in past 12 months | | |
| 1. Messman-Moore et al. (2008) | USA | 339 women | | CS | | Implied: lack of wantedness, force, threat, coercion, incapacitation | Not measured | SES (modified, SF) | | - 9.5% did report at least one rape experience, and 11.7% reported at least one experience of verbal sexual coercion (in the absence of rape) during the 8-month study (T2, T3, T4) - 88% AOD rapes; 69% AOD no force, 19% AOD force/threats, 12% threats/force no AOD  - Rape victims and verbal coercion victims reported higher levels of sexual concerns  - Rape and coercion victims had more partners and higher rates of dysfunctional sexual behaviour - Rape victims reported higher levels of dissociation and impaired self-reference, and coercion victims reported higher levels of depression, self-criticism, and impaired self-reference - Rape victims reported higher levels of alcohol use and greater endorsement of global positive and relaxation alcohol expectancies - Coerced women reported higher rates of global positive alcohol expectancies - Significant association between recent marijuana use and SV | | |
| 1. Porta et al. (2017) | USA | 6548 (F=4358, M=2057, SM=75) | | CS | | Implied against his or her will | Within SV measure | - SV perpetration was measured with two survey items (yes/no response options) - SV victimization was measured using four survey items | | - 0.8% perpetrated SV in past year - self-reported perpetrators of SV are more likely to be men (OR = 5.02), to have been a victim in his or her lifetime (OR = 3.55), to have smoked marijuana in the past 12 months (OR = 2.61), to be younger (18/19 years old; OR = 2.68), and to be Native American (OR = 16.86) or Asian (OR = 3.29) - For women, the predictive model for perpetration simplifies to a history of victimization (OR= 4.31) and use of other drugs (other than alcohol, marijuana) during the last year - Among men, significant differences were observed with higher rates among those who are Asian or American Indian compared with White, have experienced victimization, used marijuana in the past 12 months but not in the past 30 days, and used other drugs in the past 12 months  - Among women, significant differences were observed for victimization, diagnosis with alcohol and drug problems more than 12 months ago, marijuana use, and other drug use in the past 12 months | | |
| 1. Stermac et al. (2018) | Canada | 88 women | | CS | | Implied: force, threats, incapacitation | Unclear | SES-R (abbreviated) | | - Higher percentage of women with a disability (31%) reported completed sex that was unwanted following arguments and continual pressure than those with no disability (17%) - Reported odds of completed sex after receiving arguments and continual pressure were over two times that of women without disabilities - Reported a higher frequency of completed unwanted sexual acts compared to women who did not self-identify with a disability  - Completed and unwanted sex using physical force was reported by 15% of women with a disability compared to 7% for those not reporting disability - Reported odds of completed sex using physical force were two times that of women without disabilities - 22% of women with a disability reported completed sexual acts compared to 11% of women without a disability  - the reported odds of coercion while incapacitated or intoxicated were over two times more than that of women without disabilities - Women with disabilities reported sexual harassment more often than women without disabilities (61%) - the reported odds of victimization through sexual harassment were almost twice more likely than for women without disabilities | | |
| 1. Swartout et al. (2015) | USA | 184 men | | CS | | Implicit: coercion, threats, force, incapacitation | Not measured | SES | | - Perpetration: 15.2% during year 1 of college, 13.7% during year 2, and 13.1% during year 3 - Just over 31% of men reported engaging in sexually aggression at least once across the four assessments | | |
| 1. Warren et al. (2015) | USA | 217 men | | CS | | Implicit: coercion, threats, force, incapacitation | Comprehension of sexual consent/coercion scale | SES(SF-P) | | - 10% perpetrated sexual aggression in last 4 months - attachment to abusive peers had no direct effect on the hypothesized mediator, comprehension of sexual consent - Greater acceptance of rape myths, more conformity to masculine norms, and greater peer support of abuse all predicted less comprehension of sexual consent - Comprehension of sexual consent demonstrated a negative relationship to perpetration of sexual aggression; greater comprehension of sexual consent predicted lower levels of sexual perpetration - Indirect effect of RMA on perpetration of sexual aggression via comprehension of sexual consent - The indirect effect of conformity to masculine norms on perpetration of sexual aggression through comprehension of sexual consent was found - the indirect effect of peer support of abuse on perpetration of sexual aggression via comprehension of sexual consent | | |
| 1. Wong et al. (2020) | USA | 242 men | | LS | | Implicit: coercion, threats, force, incapacitation | No explicit measure | SES(SF-P) | | - Participants at T2 reported lower SPLS scores and higher BBS at T3 - ISS moderated effect of self-persuasion intervention on BBS - SPLS mediated effect of intervention - No significant results for SES | | |
| 1. Pedersen et al. (2020) | Mixed | 2630 (F= 2048, M= 573, GNC = 9) | | LS | | Implicit: coercion, threats, force, incapacitation | No explicit measure | SESV | | - 21% reported victimization abroad - mostly, non-consensual/unwanted SC (19.7%) - 1.2% experienced coercion, 0.8% experienced completed SA by force, 1.2% drug/alcohol facilitated SA and 2.4% attempted assault - Long term program, female and <21 was predictive of greater likelihood of sexual victimization - Precollege and college victimization were also predictive | | |
| 1. Johnson et al. (2020) | USA | 189 (F= 108, M= 76, GNC = 5) | | MM | | Implicit: lack of permission, lack of wantedness, force, threat, lack of willingness | No explicit measure | Open-ended questionnaire:  "While you were in college did you receive uninvited or unwanted sexual attention, such as touching, cornering, pressure for sexual favours or verbal remarks?" "While you were in college did someone ever use force or threat of force to have SC with you against your will?" | | - Sexual regret experiences were linked to Greek life, college athletics and college SV - Females endorsed greater sexual regret than males - altered judgement (*n*=16): regret due to alcohol and/or substance - Motivations (*n*=13): regretted underlying motivations for engaging in sexual activity - Partner characteristics (*n*=10): sexual regret stemming from some feature/quality of their partner - Social judgement (*n*=4): social judgements regarding partner choice and/or their character contributing to sexual regret - Unsatisfying/unpleasant sexual experiences (*n*= 2): unsatisfying or unpleasant regretted sexual experiences  - *n*=19 cases of questionable sexual consent | | |
| 1. Moschella-Smith et al. (2022) | USA | *Study 1*: 1067 (F=550, M=224, T/NB=26) *Study 2*: 1506 (F=1118, M=354, T/NB=31) | | CS | | Explicit: "the freely given verbal or nonverbal communication of a feeling of willingness to engage in sexual activity." | SCS-R | ARC3 Campus Climate Survey | | - Female, peer attitudes and knowledge of sexual consent assoc. with greater intent to help  - SV history not associated with intentions to help - Actual engagement in bystander action weakly associated with being younger, knowledge of sexual consent and SV history | | |
| 1. Marcantonio et al. (2022) | USA | 205 | | MM | | Explicit:  "one’s freely given verbal or nonverbal communication of their sober and conscious feelings of willingness to engage in a particular sexual behavior with a particular person within a particular context" | Self-report created by authors: "Were these sexual acts that happened in the past 24 hours consensual?" (Likert 7: Definitely not, no, probably not, I'm not sure, probably, yes, definitely); open text follow-up: "What was said, done or felt to make you give this rating for consent? Please be specific." - recoded into dich variable: 1-5 at least once = 1 (non-consensual), 6-7 for everything = 0 (consensual) | Self-report created by authors: "In the past 24 hours, which of these sexual behaviours have you done?" | | - 27%/26% F/M reported a non-consensual/questionably consensual experience - no gender differences in consent communication/perception - as alcohol consumption decreased, participants were 1.6x more likely to report a non-consensual/questionably consensual experience - 17.6% of those engaging in binge drinking reported at least one non-consensual/questionably-consensual experience vs 40% who did not binge drink - typical drinking was not associated with either consent communication or context; not statistically significant but binge drinking participants relied less on consent communication and more on context (than those who did not binge) | | |
| 1. Willis & Jozkowski (2019) | USA | 205 | | MM | | Explicit:  "one’s voluntary, sober, and conscious willingness to engage in a particular sexual behavior with a particular person within a particular context" | Self-report created by authors: "Were these sexual acts that happened in the past 24 hours consensual?" (Likert 7: Definitely not, no, probably not, I'm not sure, probably, yes, definitely); open text follow-up: "What was said, done or felt to make you give this rating for consent? Please be specific." | Self-report created by authors: "In the past 24 hours, which of these sexual behaviours have you done?" | | - more intimate sexual behaviours were reported less frequently for both sexual precedent (experiences with partner reported at baseline) and sexual activity (experiences reported across 30 days) reported - majority experiences were consensual - 27 experiences (probably consensual), 5 experiences (probably not consensual), 2 experiences (not consensual), 1 experience (definitely not consensual) - 16 experiences reported as not being sure whether they were consensual  - consent conceptualization refers only to consensual sexual experiences rather than both (or non-consensual) - as number of sexual behaviours with partner (sexual precedence) increased, reliance on consent comm decreased until 575 sexual behaviours; then positive relationship (as in other Willis work) - overall: university students conceptualized sexual consent communication differently depending on the magnitude of sexual history with a partner; relied more on context to rate the sexual encounter as consensual. | | |
