# Supplementary material for: Defining and Measuring Sexual Consent within the Context of University Students’ Unwanted and Nonconsensual Sexual Experiences: A Systematic Literature Review
Source: Trauma Violence Abuse. 2023 Jan 14;25(1):231–45. doi: 10.1177/15248380221147558 (PMC10666479; doi:10.1177/15248380221147558)
Supplement: sj-docx-2-tva-10.1177_15248380221147558 – Supplemental material for Defining and Measuring Sexual Consent within the Context of University Students’ Unwanted and Nonconsensual Sexual Experiences: A Systematic Literature Review [file sj-docx-2-tva-10.1177_15248380221147558.docx]

**Table 3.**

*Summary of critical findings*

**Table 4.**

*Summary of policy, practice and research implications*

| Policy | - Sexual consent definitions in sexual misconduct policies should be explicit but considerate of the student experience |
| --- | --- |
| Practice | - Sexual consent education interventions may be more effective if informed by students and their sexual consent context and understanding |
| Research | - Explicit sexual consent definitions are necessary for participants and researchers - Further research is required to explore students’ implicit sexual consent definitions - Further research is required to understand the relationship between rape myths and sexual consent conceptualization - It seems unlikely that sexual consent comprehension can be measured objectively with a Likert type scale |

| Aims | Critical findings |
| --- | --- |
| Identify a consistent definition of sexual consent within the relevant literature | - No consistent explicit definition identified but three studies referred to consent as willingness - Four themes within implicit definitions: incapacitation, use of force, use of threats and wantedness - Only two studies explored students’ individual sexual consent conceptualizations |
| Identify a reliable and consistent method of measuring sexual consent comprehension | - No discrete sexual consent comprehension measurement identified - Sexual consent typically measured implicitly via the USE measurement - Measurements primarily considered sexual consent communication or attitudes and behaviours predictive of sexual aggression |
